# Identifying and quantifying two ligand-binding sites while imaging native human membrane receptors by AFM

**DOI:** 10.1038/ncomms9857

**Published:** 2015-11-12

**Authors:** Moritz Pfreundschuh, David Alsteens, Ralph Wieneke, Cheng Zhang, Shaun R. Coughlin, Robert Tampé, Brian K. Kobilka, Daniel J. Müller

**Affiliations:** 1 Department of Biosystems Science and Engineering, Eidgenössische Technische Hochschule (ETH), Mattenstrasse 26, 4058 Basel, Switzerland; 2 Institute of Biochemistry, Biocenter, Goethe-University Frankfurt, Max-von-Laue-Strasse 9, 60438 Frankfurt/Main, Germany; 3 Department of Molecular and Cellular Physiology, Stanford University School of Medicine, 279 Campus Drive, Stanford, California 94305, USA; 4 Department of Pharmacology and Chemical Biology, University of Pittsburgh, School of Medicine, E1358 Thomas E. Starzl BST, Pittsburgh, Pennsylvania 15261, USA; 5 Cardiovascular Research Institute, University of California San Francisco, 555 Mission Bay Boulevard South, S452P, San Francisco, California 94158, USA

## Abstract

A current challenge in life sciences is to image cell membrane receptors while characterizing their specific interactions with various ligands. Addressing this issue has been hampered by the lack of suitable nanoscopic methods. Here we address this challenge and introduce multifunctional high-resolution atomic force microscopy (AFM) to image human protease-activated receptors (PAR1) in the functionally important lipid membrane and to simultaneously localize and quantify their binding to two different ligands. Therefore, we introduce the surface chemistry to bifunctionalize AFM tips with the native receptor-activating peptide and a tris-*N*-nitrilotriacetic acid (tris-NTA) group binding to a His_10_-tag engineered to PAR1. We further introduce ways to discern between the binding of both ligands to different receptor sites while imaging native PAR1s. Surface chemistry and nanoscopic method are applicable to a range of biological systems *in vitro* and *in vivo* and to concurrently detect and localize multiple ligand-binding sites at single receptor resolution.

## Abstract (web summary)

Functional analysis of membrane proteins would benefit from the combination of imaging with ligand characterisation. Here, Pfreundschuh *et al*. use specialised atomic force microscopy tips to image and quantify the binding of two ligands at the same time.

## Graphical abstract

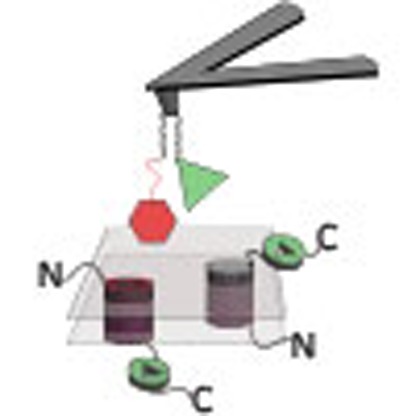

In living cells signalling processes are governed by specific extracellular and intracellular interactions. For example, extracellular ligands binding to cell surface receptors can induce conformational changes that intracellularly transduce signals^1^. In the cellular membrane, however, hundreds of different, functionally highly versatile receptors are heterogeneously distributed. Furthermore, the functional state of many receptors depends on their dynamic assembly in the cellular membrane, which means that the same type of receptor can bind a ligand strongly, weakly or not at all^2^,^3^. Moreover, because cell surface receptors can often bind a number of different ligands there is a pertinent need to image individual receptors distributed in cellular membranes and to simultaneously quantify their interaction with several ligands. However, so far there is no high-resolution (<3 nm) microscopic method that can achieve this. Such a method would be a valuable tool to characterize the diversity of ligands binding to native membrane receptors and to systematically investigate factors contributing to the functional diversity of receptors.

Force–distance curve-based atomic force microscopy (FD-based AFM) combines high-resolution imaging and single-molecule force spectroscopy^4^,^5^. While imaging a biological sample FD-based AFM approaches and retracts the AFM tip pixel by pixel. Each approach and retraction cycle is recorded in a FD curve (Supplementary Fig. 1). Analysing the FD curves provides mechanical properties about the biological sample, relating to adhesion, stiffness, deformation, elastic modulus and energy dissipation. Excitingly, functionalization of the AFM tip allows specific (bio-)chemical interactions to be detected and structurally mapped^6^,^7^. FD-based AFM methods using the AFM tip as a multifunctional toolbox have rapidly progressed in the past few years^5^,^8^. The technology is now ready to image native membrane proteins at nanometre resolution and to map their mechanical properties, electrostatic potential and chemical groups^9^,^10^,^11^. Recently, FD-based AFM was introduced to image native protein complexes at high-resolution (≈3 nm) and to structurally map their specific binding sites^12^,^13^. To be able to detect specific binding sites FD-based AFM requires tethering a ligand to the AFM tip. While contouring the protein complexes this functionalized AFM tip can then simultaneously measure the interactions of the tethered ligand to the protein. Excitingly, it was recently demonstrated that one antibody tethered to the AFM tip can be applied to distinguish between two different interactions with cell surface proteins^14^. However, so far it was not possible to image individual membrane receptors and to simultaneously detect their specific interactions with more than one ligand.

To address this challenge, we thought of imaging G-protein-coupled receptors (GPCRs) and to simultaneously quantify their binding strength to two different ligands. GPCRs are the largest family of membrane receptors and mediate most cellular responses to hormones and neurotransmitters, as well as being responsible for vision, olfaction and taste^1^,^15^,^16^,^17^. Being able to detect the binding of different ligands to individual GPCRs is of particular interest, because GPCRs even of the same type can coexist in different functional states in the cellular membrane and because most GPCRs can bind different ligands at different strength or affinity^3^,^18^. In principle we could have selected various GPCRs, including the human β_2_-adrenergic receptor, the adenosine receptor, opioid receptor, the histamine receptor or the dopamine receptor for our studies. However, we decided to focus on the human protease-activated receptor 1 (PAR1), which belongs to the class A subfamily of GPCRs and which natively binds to tethered ligands^19^,^20^,^21^. Thus, tethering this ligand to the AFM tip would enable us to mimic a native-like ligand-binding process to a GPCR. PAR1 is activated by thrombin, which cleaves part of its extracellular N-terminal domain^22^. The newly formed NH_2_-SFLLRN sequence of the N terminus acts as a tethered thrombin receptor-activating peptide. This thrombin receptor-activating peptide binds intramolecularly to PAR1, inducing a cascade of conformational changes that lead to the association of G-protein subunits. Such activation of PAR1 by the coagulation protease thrombin initiates the binding of G-proteins, triggering signalling cascades to initiate cellular responses that help to orchestrate haemostasis, thrombosis, inflammation and perhaps tissue repair^19^,^22^. Once activated, GPCRs rapidly phosphorylate, mainly by the action of G-protein-receptor kinases on serine and threonine residues localized on the third intracellular loop and C terminus^23^. PAR1 phosphorylation triggers the recruitment of arrestins, which promote the dissociation and hence, inactivation of G-protein signalling, a process referred to as desensitization.

In the following we introduce FD-based AFM to image human PAR1s in proteoliposomes and to simultaneously detect their extracellular and intracellular interactions with two ligands. As ligands we tether the native SFLLRN peptide (including the thrombin-cleaved N-terminal end) of human PAR1 and a tris-*N*-nitrilotriacetic acid (tris-NTA) group to the AFM tip. Whereas the SFLLRN peptide can bind to the extracellular ligand-binding pocket of PAR1, the tris-NTA group can bind to a His_10_-tag engineered to the intracellular C-terminal end of PAR1.

## Results

### Introducing AFM tips bifunctionalized with two ligands

**Figure 1:**
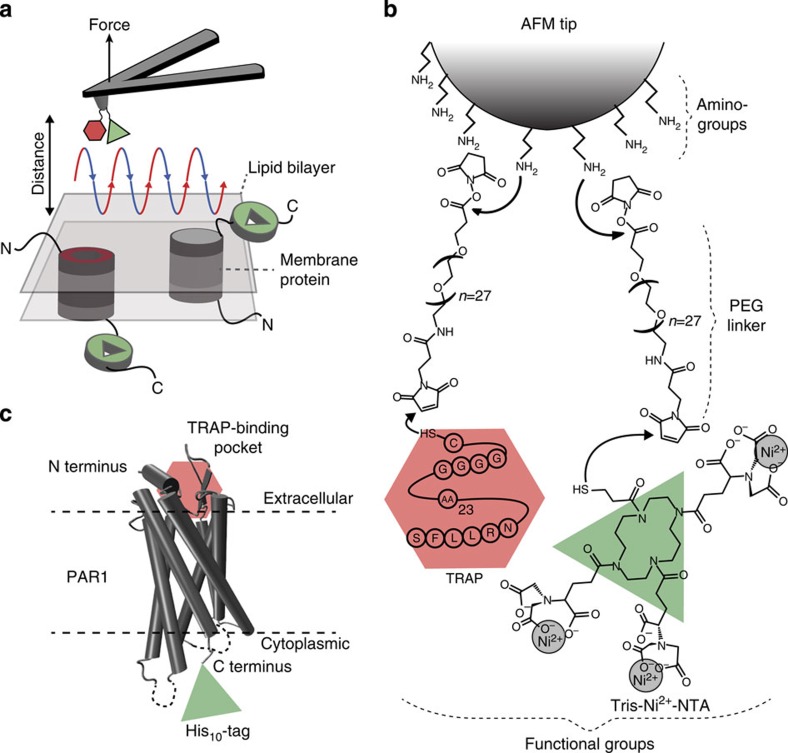
AFM mapping of two different ligand-binding events using a chemically bifunctionalized AFM tip. (**a**) Mapping adhesion forces on membrane proteins using FD-based AFM. When recording an AFM topograph an approach (blue) and retraction (red) cycle between AFM tip and biological sample is performed for every pixel of the image. In each cycle, the cantilever deflection (for example, force) and the distance travelled by the AFM tip is monitored and transformed into an approach and retraction FD curve (Supplementary Fig. 1). (**b**) Bifunctionalization of the AFM tip. The amino-functionalized Si_3_N_4_ AFM tip is functionalized by hetero-bifunctional *N*-hydroxysuccinimide-PEG_27_-maleimide linkers to which the thiol bearing thrombin receptor-activating peptide (TRAP) and Ni^2+^-loaded tris-NTA (tris-Ni^2+^-NTA) are bound. (**c**) The membrane-embedded PAR1 reveals an intracellular C-terminal His_10_-tag (green) and an extracellular TRAP (here the native SFLLRN peptide ligand)-binding pocket (red), which can specifically bind the tris-Ni^2+^-NTA and the SFLLRN ligands, respectively.

To detect specific interactions of a biological sample FD-based AFM contours the sample topography while recording for every topographic pixel at least one FD curve (Supplementary Fig. 1). On functionalizing the AFM tip with one ligand it has been shown that FD-based AFM can detect the binding events of the ligand and localize these specific interactions to the topography of the protein^12^. In our approach we wanted to contour the topography of human PAR1 proteoliposomes and to simultaneously detect the interaction of the sample with two different ligands (Fig. 1a). Thus, to simultaneously detect the binding of PAR1 to extracellular and intracellular ligands by FD-based AFM, we developed a procedure to bifunctionalize the AFM tip with two different ligands (Fig. 1b). One ligand was the native SFLLRN ligand of PAR1. To mimic as closely as possible the native binding of the ligand to PAR1 we left the SFLLRN peptide at the 28-amino acid (aa)-long N-terminal end of thrombin-cleaved PAR1. The other ligand was a tris-NTA group, which in presence of Ni^2+^ ions can interact with a His_10_-tag fused to the intracellular C terminus of the receptor (Fig. 1b–c). Whereas the tris-NTA group shows an affinity *K*_d_ to bind the His_10_-tag of ≈10–30 nM, the SFLLRN ligand shows an affinity for binding PAR1 of ≈200–800 nM (refs 24, 25, 26). To separate unspecific from specific interactions both ligands were covalently attached to a previously amino-functionalized AFM tip via a ≈10-nm-long PEG_27_ spacer^27^,^28^.

### Detecting one ligand binding after the other

**Figure 2:**
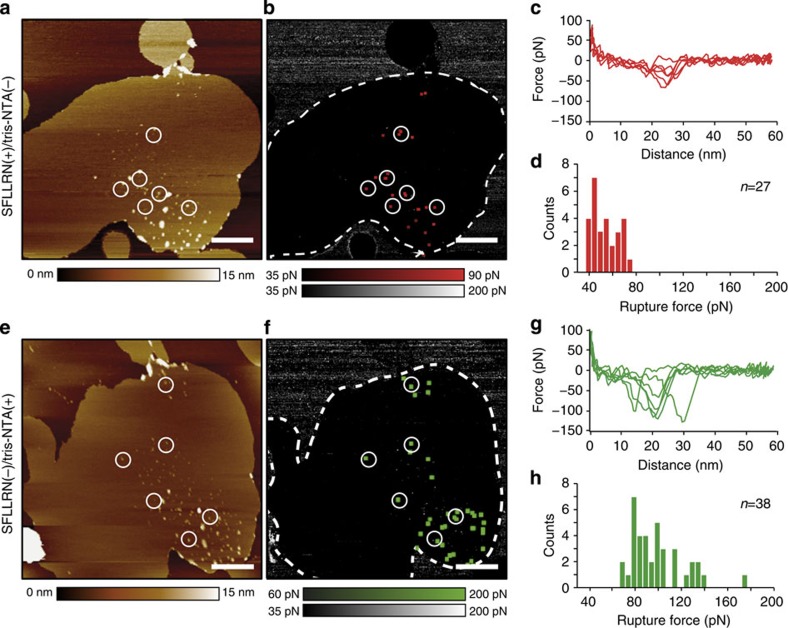
Imaging PAR1 proteoliposomes using bifunctionalized AFM tips and mapping the specific binding of SFLLRN or of tris-NTA ligands. (**a**–**d**) Imaging the proteoliposome and mapping the binding of the SFLLRN ligand to PAR1. (**a**) AFM topograph of a PAR1 proteoliposome recorded with inactivated tris-NTA (tris-NTA(−)) and active SFLLRN (SFLLRN(+)) ligand. To prevent tris-NTA interacting with the His_10_-tag of PAR1, experiments were conducted in the absence of Ni^2+^. (**b**) Adhesion map showing specific rupture forces on the proteoliposome (dashed) imaged in **a**. (**c**) FD curves recording specific interactions in **b** at distances corresponding to the length of the stretched PEG linker tethering the SFLLRN ligand to the AFM tip. (**d**) Rupture forces of single SFLLRN–PAR1 bonds range from 35 to 80 pN. (**e**–**h**) Imaging the proteoliposome and mapping the binding of the tris-NTA ligand to the His_10_-tag of PAR1. (**e**) AFM topograph of the PAR1 proteoliposome recorded with activated tris-NTA (tris-NTA(+)) and blocked SFLLRN (SFLLRN(−)) ligand. To promote tris-NTA binding to the His_10_-tag and to prevent the SFLLRN ligand binding to PAR1 the experiments were conducted in the presence of Ni^2+^ and the antagonist BMS. (**f**) Adhesion map showing specific rupture forces on the proteoliposome (dashed) imaged in **e**. (**g**) FD curves recording specific interactions in **f**. (**h**) Rupture forces of single tris-NTA–His_10_-tag bonds range from 65 to 140 pN. The encircled positions of AFM topographs and adhesion maps indicate where specific interactions were detected. Topographic heights (**a** and **e**) are indicated by colour bars. Rupture force distributions (**c**,**d**,**g**,**h**) shown at 5 pN bin size. Images were recorded in 300 mM NaCl, 20 mM HEPES, 25 mM MgCl_2_, pH 7.2 and if stated 2 μM BMS or/and 5 mM NiCl_2_. Experiments were repeated six times, each time we prepared a new sample and used different functionalized AFM tips. Scale bars, 500 nm (**a**,**e**).

After bifunctionalizing the AFM tip, we adsorbed PAR1 proteoliposomes to freshly cleaved mica and imaged the sample in buffer solution by FD-based AFM using the bifunctionalized tip (Fig. 2). To first detect only the interaction of the SFLLRN ligand to PAR1, we used imaging buffer lacking Ni^2+^ ions, which are needed to coordinate the tris-NTA to the His_10_-tag. We recorded FD-based AFM topographs with 256 × 256 pixels (2.5 × 2.5 μm^2^) and one approach and one retraction FD curve per pixel. The exemplified topograph precisely showed the lipid membrane with protrusions corresponding to single and assemblies of several PAR1s (Fig. 2a and Supplementary Fig. 2). The single-layered lipid membrane indicated that on adsorption to mica the proteoliposome opened. Analysing the adhesion detected in each FD curve recorded in the topograph allowed to calculate the adhesion map (Fig. 2b). Correlation of this adhesion map with the topograph localized unspecific interactions of the AFM tip with the supporting mica and specific interactions with the receptors. Characteristic adhesive forces arising from specific interactions were detected at tip-sample distances >7 nm, which correlates to the length of the extended PEG_27_ linker (≈10 nm) and of the 28-aa-long native N-terminal sequence of PAR1 tethering the SFLLRN ligand to the tip (Fig. 2c and Supplementary Note 1). The variation of the distances at which the rupture forces were detected resulted from SFFLRN ligands being attached at different positions to the AFM tip (Supplementary Fig. 3). In rare cases (<5%), rupture forces were detected at distances longer than the linker system, suggesting that the mechanical force applied to the receptor lifted the soft lipid membrane^29^. Such force curves were excluded from analysis (Supplementary Note 1). As a further control, we validated the extension profile of every single-adhesion peak detected in a FD curve by fitting the polyethylene glycol (PEG)-polypeptide linker system attaching the SFLLRN ligand to the AFM tip (Supplementary Figs 3,4 and 7). The forces of these adhesion peaks ranged from 35 to 80 pN (Fig. 2d), which is the typical force required to separate ligand–receptor bonds^30^,^31^,^32^,^33^. In several controls, we showed that non-functionalized AFM tips, AFM tips functionalized with only the PEG_27_ spacer and AFM tips functionalized with the scrambled SFLLRN peptide LFRLSN did not detect specific interactions with the supporting mica or with the PAR1 proteoliposome (Supplementary Figs 5 and 6).

After having detected the binding of the extracellular SFLLRN ligand, we wanted to detect only the intracellular His_10_ tags fused to the C terminus of the receptor. To promote binding of tris-NTA to the His_10_-tag, we pre-incubated the bifunctionalized AFM tip with 10 mM NiCl_2_ and added 5 mM NiCl_2_ to the imaging buffer. To prevent binding of the SFLLRN peptide to PAR1, we added the PAR1 antagonist BMS-200261 (BMS)^26^ to the buffer solution. Subsequently, FD-based AFM was used to image the same PAR1 proteoliposome as imaged before for SFLLRN binding (Fig. 2e). It is important to note, that the proteoliposome had a slightly different shape due to membrane mobility. Again, the adhesion map showed unspecific interactions of the bifunctionalized AFM tip with the supporting mica and localized specific interactions to PAR1s imaged in the topograph (Fig. 2f). Specific interactions were detected at tip-sample distances >7 nm, which correlated to the length of the stretched bifunctional PEG_27_ linker and of the stretched C-terminal end of PAR1 to which the His_10_-tag has been fused (Fig. 2g and Supplementary Fig. 3)^29^,^34^. As a further control, we validated the extension profile of every single-adhesion peak detected in a FD curve by fitting the PEG_27_-polypeptide linker system attaching the tris-Ni^2+^-NTA ligand to the AFM tip (Supplementary Figs 3,4 and 7). The forces of these adhesion peaks ranged from 65 to 250 pN (Fig. 2h), which is the characteristic force required to separate the bond formed between tris-NTA and His_10_-tag^24^,^35^,^36^.

### Blocking specific interactions of the bifunctionalized tip

Next, we tested the specificity of our bifunctionalized AFM tip by blocking all specific interactions to the PAR1 proteoliposome. To do this, we left the antagonist BMS in the buffer solution and removed Ni^2+^ by adding 10 mM EDTA, and imaged the proteoliposome using FD-based AFM (Supplementary Fig. 8). The functionalized AFM tip showed unspecific interactions with mica and few unspecific interactions with the proteoliposome. Very rarely, force curves (≈1–2 per adhesion map of 256 × 256 (65,536) curves) showed the signature of a ‘specific interaction'. We attributed these interaction events to rupture forces of tip-tethered SFLLRN peptides bound to single PAR1s. For these specific ligand-binding events, one must assume that, having a dissociation constant of ≈425 nM (ref. 26), single PAR1s spontaneously dissociated from the BMS antagonist. Alternatively, in rare cases BMS may have dissociated from PAR1 while the receptor was being mechanically probed by the AFM tip. However, independent of how rare binding cases may have occurred, this experiment nicely highlights that the high-affinity antagonist BMS inhibits binding of the tethered SFLLRN ligand to PAR1 (ref. 13). In summary, these control experiments demonstrate that the antagonist and removal of Ni^2+^ successfully blocked both ligands tethered to the AFM tip. The experiments further confirm that the AFM tip was functionalized with both ligands.

### Simultaneously detecting two different specific interactions

**Figure 3:**
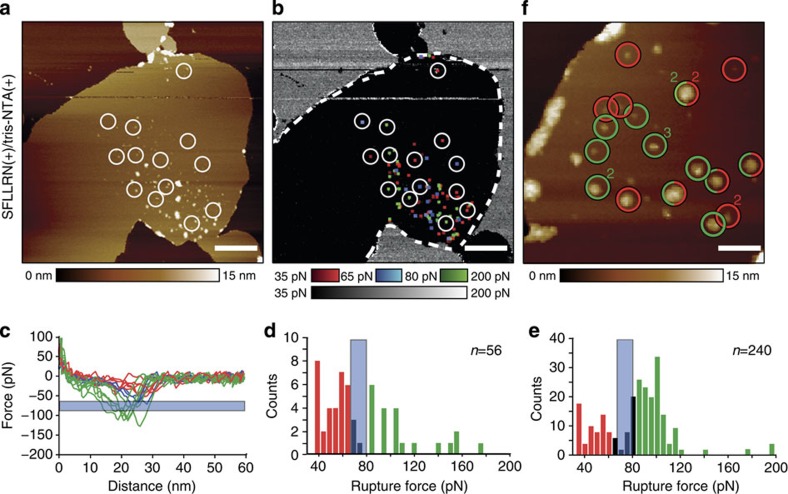
Mapping the binding of two different ligands while imaging PAR1 proteoliposomes. (**a**) AFM topograph of a PAR1 proteoliposome. Measurements were performed in the presence of Ni^2+^ to allow both tip-tethered ligands, the tris-NTA and the SFLLRN peptide to interact with PAR1-binding sites. (**b**) Adhesion map simultaneously recorded with the topograph (**a**). Adhesion events detected on the proteoliposome (dashed boundary) categorize into three different interaction force scales: 30–65 pN for the extracellular SFLLRN–PAR1 bond (red), 65–80 pN for the force filter applied (blue) and 80–200 pN for the intracellular tris-Ni^2+^-NTA–His_10_-tag bond (green). (**c**) Selected FD curves showing specific adhesion forces; recorded at areas encircled in **a**,**b**. (**d**) Distribution of forces characterizing the rupture of ligand–receptor bonds. Applying a force filter from 65 to 80 pN (blue) discriminates between both types of specific interactions (red versus green). (**e**) Distribution of rupture forces of SFLLRN–PAR1 (red) and of tris-NTA–His_10_-tag (green) bonds recorded in three consecutive images of the PAR1 proteoliposome. (**f**) PAR1 proteoliposome imaged at higher magnification. Specific interaction sites detected after three consecutive recordings were encircled and colour coded for extracellular SFLLRN–PAR1 (red circles) and intracellular tris-NTA–His_10_-tag (green circles) interactions. The numbers two and three indicate that the specific interactions were detected two or three times, respectively. Topographic heights (**a**,**e**) are indicated by colour bars. Rupture force distributions (**c**,**d**,**f**) shown at 5 pN bin size. Images were recorded in 300 mM NaCl, 20 mM HEPES, 25 mM MgCl_2_, 5 mM NiCl_2_, pH 7.2. Experiments were repeated six (**a**,**b**) or three (**f**) times, each time using new sample preparations and functionalized AFM tips. Scale bars, 500 nm (**a**) and 80 nm (**f**).

After having shown that the bifunctionalized AFM tip can either detect the His_10_-tag or the ligand-binding site of PAR1, we wanted to investigate whether we could use the tip to simultaneously detect both specific interactions. Therefore, FD-based AFM topographs were recorded in physiological buffer solution supplemented with 5 mM NiCl_2_ (Fig. 3a), which allows both ligands of the bifunctionalized tip to detect specific interactions with PAR1. The adhesion map showed specific interactions with ≈43% (*n*=56) of the PAR1s topographically imaged (Fig. 3b). After imaging the sample three consecutive times, ≈80% of the PAR1 imaged showed specific interactions with the tethered ligands. These interactions were detected at rupture lengths corresponding to the different PEG_27_-polypeptide linker systems (Fig. 3c). To distinguish between the rupture events of SFLLRN and tris-NTA ligands we set-up a force filter. As measured above, the strength of the SFLLRN–PAR1 bond ranges from 35 to 80 pN, whereas that of the tris-NTA–His_10_-tag bond ranges from 65 to 250 pN (Fig. 2 and Supplementary Fig. 9). We thus designed the force filter to discard all rupture events between 65 and 80 pN (Fig. 3d,e). Accordingly, adhesive forces <65 pN were allocated to SFLLRN–PAR1 bonds and forces >80 pN to tris-NTA–His_10_-tag bonds.

To further increase the probability of detecting ligand binding by all receptors we repeatedly imaged the same proteoliposome at a ≈3.5-fold higher magnification, thereby increasing the number of FD curves (pixels) probed per PAR1. After three consecutive recordings, we observed specific ligand-binding events for ≈90% of the PAR1s. On the basis of the strength of the unbinding events detected, we could assign which of the two ligands bound and, as one binds to the extracellular and the other to the intracellular surface of individual PAR1s, determine the orientation of the reconstituted receptors in the membrane. In larger assemblies of PAR1s we could observe receptors in both orientations (Fig. 3f and Supplementary Fig. 10). This is not surprising since membrane proteins reconstituted into symmetric lipid membranes often show mixed orientations. Although being repeatedly imaged some of the PAR1 receptors neither bound the SFLLRN ligand nor tris-NTA (Fig. 3). One may argue that one must simply increase the number of images taken until these receptors bind the ligand. However, GPCRs in the unbound state or ligand-bound state can reside in different functional states or conformations^3^,^18^. Some of these functional states can bind a particular ligand while others cannot. Thus, one may speculate that the PAR1 molecule to which the ligands cannot bind resides in an inactive state. It may be interesting in the future to characterize the parameters (for example, pH, electrolyte, drugs, proteins and lipids) modulating these conformational states similar to what we observed on addition of the antagonist BMS (Fig. 2). Compared with multicolour fluorescence microscopy imaging, our approach shows that even two distinct functional groups/proteins can be detected and localized at separations ≤5 nm. Such differential imaging of membrane receptors binding to different ligands will be a useful tool to investigate the composition and functional state of membrane–protein complexes in their native environment at unprecedented detail (resolution) and information (binding strength).

## Discussion

Here we have introduced the high-resolution (≈3 nm) imaging of a native human GPCR, PAR1, embedded in a functionally relevant membrane environment and simultaneously detected the specific binding of two different ligands. To achieve this goal, we bifunctionalized the AFM tip with one ligand interacting with the extracellular binding site of PAR1 and another ligand interacting with the intracellular surface of PAR1. Using FD-based AFM we imaged PAR1 proteoliposomes and localized specific ligand-binding forces to PAR1s. The introduction of a force filter discriminated between the two specific ligand–receptor bonds and enabled us to classify individual receptors according to the ligand bound. In principle our bifunctionalization approach is not restricted to reconstituted membrane receptors and applicable to force probe a wide variety of biological systems and map their interactions with different ligands or chemical groups at the same time. For example, it has been demonstrated that FD-based AFM can be readily applied to detect specific interactions of individual surface receptors in living cells^10^,^33^,^37^. Thus, the here-introduced approach to functionalize the AFM tip with two ligands may be applied to image living cells by AFM and to simultaneously map two different cell surface molecules.

We have applied FD-based AFM to image a GPCR and to measure the forces required to rupture single receptor–ligand bonds. At first sight, these forces cannot be directly related to describe the thermodynamic and kinetic properties of the bond. However, theoretical procedures have been recently introduced, which allow extrapolating the parameters from FD-based AFM data to describe these thermodynamic and kinetic properties^38^,^39^,^40^. Therefore, the rupture forces must be determined over a wide range of pulling velocities. Recently, an experimental approach has been introduced, which applies these theories to extract these parameters from FD-based AFM experiments and to reconstruct the ligand-binding free-energy landscape of GPCRs^13^. It appears to be a matter of time until this and our approach introduced here can be combined to image GPCRs in cellular membranes and to simultaneously determine their free-energy landscape with more than only one ligand. Such a combined approach may help us in the future to learn understanding the conditions at which a GPCR prefers to bind one ligand against the other.

We here used our approach to differentiate between two ligands having different binding strengths to PAR1. But how to differentiate between two linkers binding at the same strength to receptors? The force at which a ligand–receptor bond ruptures depends on the loading rate applied^31^,^40^,^41^,^42^. Thus, to overcome such challenge linker systems having mechanically very different properties may be chosen to apply different loading rates to the individual ligand–receptor bonds and thereby to shift the rupture forces of both bonds to different values. Alternatively, however, one may simply use linker systems having very different lengths to discern between the binding of two different ligands. Taking such or similar approaches and together with a better force resolution, one may in the future even consider increasing the number of ligands that can be simultaneously characterized while imaging single receptors *in vitro* and *in vivo*.

## Methods

### Preparation and reconstitution of PAR1

Wild-type PAR1 was generated with an N-terminal FLAG epitope and 10 histidines (His_10_-tag) at the C-terminal end to facilitate protein purification. The C terminus of PAR1 was truncated after residue Tyr397, and the N terminus started from residue Ala36. This construct was tested in cell-based assays to ensure that signalling still occurred in response to thrombin activation. PAR1 was expressed in Sf9 cells (Expression Systems) using the pFastBac baculovirus system (Invitrogen). To purify PAR1, infected cells were lysed by osmotic shock in low-salt buffer containing 10 mM Tris-HCl (pH 7.5), 1 mM EDTA, 100 nM vorapaxar derivative and 100 μM tris-(2-carboxyethyl)phosphine (TCEP). The vorapaxar derivative generated by reducing the non-aromatic carbon–carbon double bond in vorapaxar showed a much faster dissociation rate than vorapaxar in cell-based assays. PAR1 was further extracted from cell membranes with buffer comprised of 20 mM HEPES (pH 7.5), 500 mM NaCl, 1% dodecyl maltoside (DDM), 0.03% cholesterol hemisuccinate (CHS), 0.2% sodium cholate, 15% glycerol, 100 nM vorapaxar derivative and 100 μM TCEP. Cell debris was removed by high-speed centrifugation. From this point on, 1 μM vorapaxar derivative was added to all buffers used for the purification, except for the buffer used for the size-exclusion chromatography. Ni^2+^-NTA agarose resin was added to the supernatant after homogenization and stirred for 1 h at 4 °C. The resin was then washed three times in batch with buffer comprised of 20 mM HEPES (pH 7.5), 500 mM NaCl, 0.1% DDM, 0.02% CHS and 1 μM vorapaxar derivative, and transferred to a glass column. Bound receptor was eluted with buffer containing 300 mM imidazole and loaded onto an anti-FLAG M1 affinity column. After extensive washing with buffer comprised of 20 mM HEPES (pH 7.5), 500 mM NaCl, 0.1% DDM, 0.02% CHS, 1 μM vorapaxar derivative and 2 mM Ca^2+^, the receptor was eluted from M1 resin using the same buffer without Ca^2+^ but with 200 μg ml^−1^ FLAG peptide and 5 mM EDTA. Size-exclusion chromatography was employed to obtain the final monodisperse receptor preparation. The running buffer contained 20 mM HEPES (pH 7.5), 100 mM NaCl, 0.1% DDM and 0.02% CHS. The flow rate was set at 0.2 ml min^−1^ to allow enough time for the vorapaxar derivative to dissociate from the receptor. The purified unliganded PAR1 was then reconstituted at 10 μM in liposomes made of 0.5 mg ml^−1^ phospholipids (1,2-dioleoyl-*sn*-glycero-3-phosphocholine) and 0.05 mg ml^−1^ of the cholesterol analogue CHS^43^. Vorapaxar-bound PAR1 was prepared as described above except that 100 nM vorapaxar, not its derivative, was used for the lysis and solubilization of cell membranes and 1 μM vorapaxar was used in the following Ni^2+^-NTA, anti-FLAG M1 affinity and size-exclusion chromatography steps.

### AFM tip functionalization

To functionalize the Si_3_N_4_ tips of the AFM cantilevers (Biolever Mini, Olympus)^28^,^44^, they were cleaned with ethanol (10 min), dried with filtered N_2_ and treated with an ultraviolet radiation and ozone cleaner (Jetlight, CA, USA) for 10 min. For the amino-functionalization, AFM tips were immersed in an 8.1 M ethanolamine solution (ethanol in dimethylsulphoxide) for 16 h. Tips were cleaned 3 × 1 min in dimethylsulphoxide and 2 min in ethanol, rinsed with ethanol and dried with filtered N_2_. For the linker attachment, the tips were immersed in a solution of maleimide–PEG_27_–*N*-hydroxysuccinimide (1 mg dissolved in 0.5 ml CHCl_3_) and 30 μl triethylamine for 2 h. Then, tips were cleaned 3 × 10 min in CHCl_3_ and dried with filtered N_2_. A volume of 50 μl of 100 μM of each of the SH-group-bearing functional groups was premixed with 2 μl of 1 M EDTA, 5 μl of 1 M HEPES (pH 7.5), 2 μl of 100 mM TCEP hydrochloride and 2 μl of 1 M HEPES (pH 9.0). This mixture was pipetted onto the cantilevers. After a reaction time of 4 h, the cantilevers were washed in PBS (3 × 5 min) and used within 24 h. Tips functionalized with the tris-NTA group were immersed in 5 mM NiCl_2_ for 10 min before use. The tris-NTA-thiol compound (mercapto propionic acid tris-NTA) was synthesized following published procedures^35^,^45^. The cysteine bearing peptide (NH_2_–SFLLRNPNDKYEPFWEDEEKNESGLTEYRGGGGC–COOH) was purchased (GenSript, USA).

### PAR1 preparation for AFM imaging

A 300-fold diluted solution in imaging buffer (300 mM NaCl, 20 mM HEPES and 25 mM MgCl_2_) of PAR1 reconstituted in liposomes was adsorbed to freshly cleaved mica for 1 h. The adsorbed sample was rinsed with the same buffer five times before AFM imaging in imaging buffer.

### FD-based AFM

A Multimode8 AFM with a Nanoscope5 controller (Bruker, Santa Barbara, California, USA) was operated in the ‘PeakForceTapping' mode. The AFM was equipped with a 120-μm piezoelectric scanner (J scanner). Rectangular Si_3_N_4_ cantilevers with nominal spring constants of ≈0.04–0.08 N m^−1^ and a resonance frequency of ≈35 kHz in water were chosen (Biolever Mini, Olympus). All AFM experiments were done in imaging buffer solution at room temperature (≈26 °C). Overview images (2–25 μm) were recorded at 2 kHz oscillation frequency, applying an imaging force of 100–200 pN, scanned at 1 line per s, with a vertical oscillation amplitude of 15 nm and a resolution of 512 × 512 pixels. Adhesion maps were recorded by oscillating the functionalized tip at 0.25 kHz, with an amplitude of 30 nm, and applying an imaging force of 100 pN. Topographs of 256 × 256 pixels were recorded scanning 0.125 line per s. The contact time between tip and sample was ≈1 ms, which corresponds to about one quarter of the oscillation cycle.

### Data analysis

The force versus time curves of each interaction recognition experiment were saved and exported as ASCII files. The Matlab software was used to translate force versus time curves into FD curves. FD curves showing specific adhesion events were analysed further. FD curves showing adhesion events at distances much longer than that of the linker system (>>30 nm) were discarded from analysis (Supplementary Note 1). Correlation of topograph and adhesion map allowed assigning the specific adhesion events to individual PAR1s. For better visibility pixels detecting adhesion events have been enlarged.

### Control experiments

We performed several control experiments to test whether the functionalization of the AFM tip was successful and whether nonspecific interactions were detected with the sample. Therefore, we imaged the proteoliposomes with an unmodified (Si_3_N_4_) AFM tip and recorded adhesion maps (Supplementary Fig. 5a–d). Furthermore, we imaged the sample with a PEG_27_-maleimide functionalized tip having no functional groups attached to the terminal maleimide group (Supplementary Fig. 5e–h) and having the scrambled SFLLRN peptide LFRLSN attached (Supplementary Fig. 6). In addition, we tested ligand binding in the absence and presence of the full antagonist BMS (Supplementary Fig. 8).

## Additional information

**How to cite this article:** Pfreundschuh, M. *et al*. Identifying and quantifying two ligand-binding sites while imaging native human membrane receptors by AFM. *Nat. Commun.* 6:8857 doi: 10.1038/ncomms9857 (2015).

## Supplementary Material

Supplementary Information Supplementary Figures 1-10, Supplementary Note 1 and Supplementary References

## References

[b1] Venkatakrishnan A. J. . Molecular signatures of G-protein-coupled receptors. Nature 494, 185–194 (2013). 23407534 10.1038/nature11896

[b2] Rajendran L., Knolker H. J. & Simons K. Subcellular targeting strategies for drug design and delivery. Nat. Rev. Drug Discov. 9, 29–42 (2010). 20043027 10.1038/nrd2897

[b3] Kobilka B. K. & Deupi X. Conformational complexity of G-protein-coupled receptors. Trends Pharmacol. Sci. 28, 397–406 (2007). 17629961 10.1016/j.tips.2007.06.003

[b4] Heinz W. F. & Hoh J. H. Spatially resolved force spectroscopy of biological surfaces using the atomic force microscope. Trends Biotechnol. 17, 143–150 (1999). 10203772 10.1016/s0167-7799(99)01304-9

[b5] Dufrene Y. F., Martinez-Martin D., Medalsy I., Alsteens D. & Muller D. J. Multiparametric imaging of biological systems by force-distance curve-based AFM. Nat. Methods 10, 847–854 (2013). 23985731 10.1038/nmeth.2602

[b6] Frisbie C. D., Rozsnyai L. F., Noy A., Wrighton M. S. & Lieber C. M. Functional group imaging by chemical force microscopy. Science 265, 2071–2074 (1994). 17811409 10.1126/science.265.5181.2071

[b7] Hinterdorfer P. & Dufrene Y. F. Detection and localization of single molecular recognition events using atomic force microscopy. Nat. Methods 3, 347–355 (2006). 16628204 10.1038/nmeth871

[b8] Muller D. J. & Dufrene Y. F. Atomic force microscopy as a multifunctional molecular toolbox in nanobiotechnology. Nat. Nanotechnol. 3, 261–269 (2008). 18654521 10.1038/nnano.2008.100

[b9] Medalsy I., Hensen U. & Muller D. J. Imaging and quantifying chemical and physical properties of native proteins at molecular resolution by force-volume AFM. Angew. Chem. Int. Ed. 50, 12103–12108 (2011). 10.1002/anie.201103991 22006839

[b10] Alsteens D. . High-resolution imaging of chemical and biological sites on living cells using peak force tapping atomic force microscopy. Langmuir 28, 16738–16744 (2012). 23198968 10.1021/la303891j

[b11] Pfreundschuh M., Hensen U. & Muller D. J. Quantitative imaging of the electrostatic field and potential generated by a transmembrane protein pore at subnanometer resolution. Nano Lett. 13, 5585–5593 (2013). 24079830 10.1021/nl403232z

[b12] Pfreundschuh M., Alsteens D., Hilbert M., Steinmetz M. O. & Muller D. J. Localizing chemical groups while imaging single native proteins by high-resolution atomic force microscopy. Nano Lett. 14, 2957–2964 (2014). 24766578 10.1021/nl5012905

[b13] Alsteens D. . Imaging G protein-coupled receptors while quantifying their ligand-binding free-energy landscape. Nat. Methods 12, 845–851 (2015). 26167642 10.1038/nmeth.3479 PMC5087271

[b14] Alsteens D., Garcia M. C., Lipke P. N. & Dufrene Y. F. Force-induced formation and propagation of adhesion nanodomains in living fungal cells. Proc. Natl Acad. Sci. USA 107, 20744–20749 (2010). 21059927 10.1073/pnas.1013893107 PMC2996417

[b15] Palczewski K. G protein-coupled receptor rhodopsin. Annu. Rev. Biochem. 75, 743–767 (2006). 16756510 10.1146/annurev.biochem.75.103004.142743 PMC1560097

[b16] Rosenbaum D. M., Rasmussen S. G. & Kobilka B. K. The structure and function of G-protein-coupled receptors. Nature 459, 356–363 (2009). 19458711 10.1038/nature08144 PMC3967846

[b17] Granier S. & Kobilka B. A new era of GPCR structural and chemical biology. Nat. Chem. Biol. 8, 670–673 (2012). 22810761 10.1038/nchembio.1025 PMC4031315

[b18] Deupi X. & Kobilka B. K. Energy landscapes as a tool to integrate GPCR structure, dynamics, and function. Physiology (Bethesda) 25, 293–303 (2010). 20940434 10.1152/physiol.00002.2010 PMC3056154

[b19] Coughlin S. R. Thrombin signalling and protease-activated receptors. Nature 407, 258–264 (2000). 11001069 10.1038/35025229

[b20] Adams M. N. . Structure, function and pathophysiology of protease activated receptors. Pharmacol. Ther. 130, 248–282 (2011). 21277892 10.1016/j.pharmthera.2011.01.003

[b21] Hollenberg M. D. . Biased signalling and proteinase-activated receptors (PARs): targeting inflammatory disease. Br. J. Pharmacol. 171, 1180–1194 (2014). 24354792 10.1111/bph.12544 PMC3952797

[b22] Coughlin S. R. Protease-activated receptors in hemostasis, thrombosis and vascular biology. J. Thromb. Haemost. 3, 1800–1814 (2005). 16102047 10.1111/j.1538-7836.2005.01377.x

[b23] Moore C. A., Milano S. K. & Benovic J. L. Regulation of receptor trafficking by GRKs and arrestins. Annu. Rev. Physiol. 69, 451–482 (2007). 17037978 10.1146/annurev.physiol.69.022405.154712

[b24] Lata S., Reichel A., Brock R., Tampe R. & Piehler J. High-affinity adaptors for switchable recognition of histidine-tagged proteins. J. Am. Chem. Soc. 127, 10205–10215 (2005). 16028931 10.1021/ja050690c

[b25] Vassallo R. R. Jr, Kieber-Emmons T., Cichowski K. & Brass L. F. Structure-function relationships in the activation of platelet thrombin receptors by receptor-derived peptides. J. Biol. Chem. 267, 6081–6085 (1992). 1313429

[b26] Bernatowicz M. S. . Development of potent thrombin receptor antagonist peptides. J. Med. Chem. 39, 4879–4887 (1996). 8960546 10.1021/jm960455s

[b27] Barattin R. & Voyer N. Chemical modifications of AFM tips for the study of molecular recognition events. Chem. Commun. (Camb.) 13, 1513–1532 (2008). 18354789 10.1039/b614328h

[b28] Wildling L. . Probing binding pocket of serotonin transporter by single molecular force spectroscopy on living cells. J. Biol. Chem. 287, 105–113 (2012). 22033932 10.1074/jbc.M111.304873 PMC3249061

[b29] Kocun M. & Janshoff A. Pulling tethers from pore-spanning bilayers: towards simultaneous determination of local bending modulus and lateral tension of membranes. Small 8, 847–851 (2012). 22228680 10.1002/smll.201101557

[b30] Florin E.-L., Moy V. T. & Gaub H. E. Adhesion forces between individual ligand-receptor pairs. Science 264, 415–417 (1994). 8153628 10.1126/science.8153628

[b31] Evans E. A. & Calderwood D. A. Forces and bond dynamics in cell adhesion. Science 316, 1148–1153 (2007). 17525329 10.1126/science.1137592

[b32] Helenius J., Heisenberg C. P., Gaub H. E. & Muller D. J. Single-cell force spectroscopy. J. Cell Sci. 121, 1785–1791 (2008). 18492792 10.1242/jcs.030999

[b33] Muller D. J., Helenius J., Alsteens D. & Dufrene Y. F. Force probing surfaces of living cells to molecular resolution. Nat. Chem. Biol. 5, 383–390 (2009). 19448607 10.1038/nchembio.181

[b34] Zocher M., Zhang C., Rasmussen S. G., Kobilka B. K. & Muller D. J. Cholesterol increases kinetic, energetic, and mechanical stability of the human beta2-adrenergic receptor. Proc. Natl Acad. Sci. USA 109, E3463–E3472 (2012). 23151510 10.1073/pnas.1210373109 PMC3528585

[b35] Valiokas R. . Self-assembled monolayers containing terminal mono-, bis-, and tris-nitrilotriacetic acid groups: characterization and application. Langmuir 24, 4959–4967 (2008). 18393558 10.1021/la703709a

[b36] Tang J. . Detection of metal binding sites on functional S-layer nanoarrays using single molecule force spectroscopy. J. Struct. Biol. 168, 217–222 (2009). 19232541 10.1016/j.jsb.2009.02.003

[b37] Alsteens D., Trabelsi H., Soumillion P. & Dufrene Y. F. Multiparametric atomic force microscopy imaging of single bacteriophages extruding from living bacteria. Nat. Commun. 4, 2926 (2013). 24336094 10.1038/ncomms3926

[b38] Evans E. Energy landscapes of biomolecular adhesion and receptor anchoring at interfaces explored with dynamic force spectroscopy. Faraday Discuss. 111, 1–16 (1998). 10822596 10.1039/a809884k

[b39] Dudko O. K., Hummer G. & Szabo A. Theory, analysis, and interpretation of single-molecule force spectroscopy experiments. Proc. Natl Acad. Sci. USA 105, 15755–15760 (2008). 18852468 10.1073/pnas.0806085105 PMC2572921

[b40] Friddle R. W., Noy A. & De Yoreo J. J. Interpreting the widespread nonlinear force spectra of intermolecular bonds. Proc. Natl Acad. Sci. USA 109, 13573–13578 (2012). 22869712 10.1073/pnas.1202946109 PMC3427124

[b41] Evans E. & Ritchie K. Dynamic strength of molecular adhesion bonds. Biophys. J. 72, 1541–1555 (1997). 9083660 10.1016/S0006-3495(97)78802-7 PMC1184350

[b42] Dudko O. K., Hummer G. & Szabo A. Intrinsic rates and activation free energies from single-molecule pulling experiments. Phys. Rev. Lett. 96, 108101 (2006). 16605793 10.1103/PhysRevLett.96.108101

[b43] Zocher M., Fung J. J., Kobilka B. K. & Muller D. J. Ligand-specific interactions modulate kinetic, energetic, and mechanical properties of the human beta2 adrenergic receptor. Structure 20, 1391–1402 (2012). 22748765 10.1016/j.str.2012.05.010 PMC4506644

[b44] Wildling L. . Linking of sensor molecules with amino groups to amino-functionalized AFM tips. Bioconjug. Chem. 22, 1239–1248 (2011). 21542606 10.1021/bc200099t PMC3115690

[b45] Tinazli A. . High-affinity chelator thiols for switchable and oriented immobilization of histidine-tagged proteins: a generic platform for protein chip technologies. Chemistry 11, 5249–5259 (2005). 15991207 10.1002/chem.200500154

