# Supplementary material for: Identifying and quantifying two ligand-binding sites while imaging native human membrane receptors by AFM
Source: Nat Commun. 2015 Nov 12;6:8857. doi: 10.1038/ncomms9857 (PMC4660198; doi:10.1038/ncomms9857)
Supplement: Supplementary Information — Supplementary Figures 1-10, Supplementary Note 1 and Supplementary References [file ncomms9857-s1.pdf]

Supplementary Information

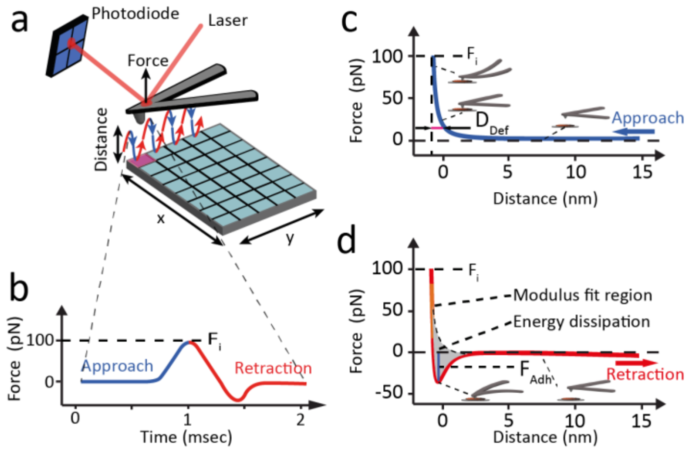

Supplementary Figure 1 | Force-distance curve-based (FD-based) AFM. (**a**) To contour the surface of the sample FD-based AFM approaches and retracts the tip of the AFM cantilever pixel-by-pixel. For each pixel the maximum cantilever deflection and thus the imaging force F_i (here limited to 100 pN) is controlled. (**b**) Force *vs* time curves of the approach (blue) and retraction (red) movements are recorded for each pixel. (**c**) Mechanical parameters such as the sample deformation D_{Def} can be extracted from the approach force *vs* distance (FD) curve (blue). (**d**) Adhesion force F_{adh}, Young's modulus, and energy dissipation can be extracted from the retraction FD curve (red). The adhesion force measures attractive interactions between the AFM tip and the sample. (**e**) The sample topography is reconstructed from the vertical AFM tip movement needed to reach the imaging force F_i for each pixel. From FD curves recorded for each pixel multiple parameters including adhesion and deformation can be determined and directly mapped to the topograph.

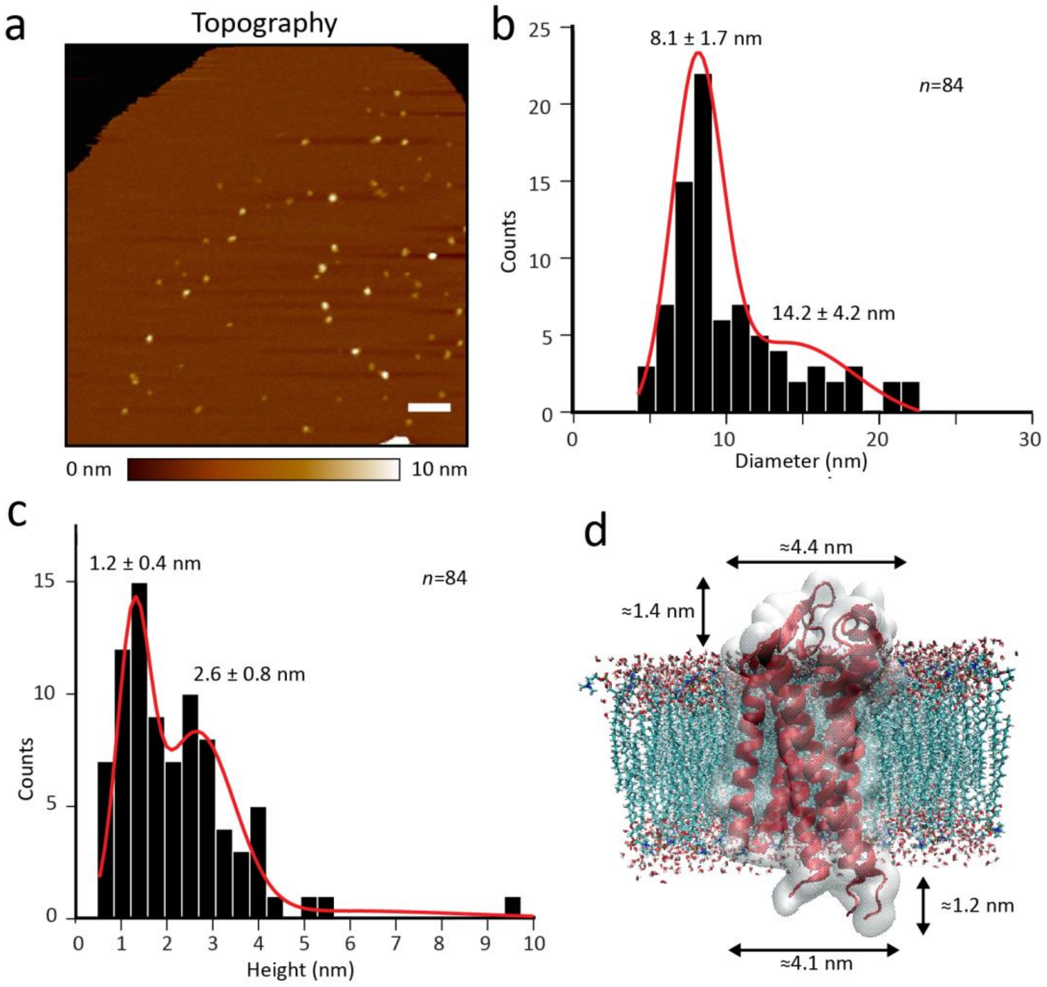

Supplementary Figure 2 | Topological analysis of human protease activated receptor 1 (PAR1) reconstituted in proteoliposomes. (a) Topography of PAR1s sparsely distributed in lipid membranes made of 0.5 mg mL^{-1} DOPC and 0.05 mg mL^{-1} CHS. Note that after adsorption to the supporting mica the proteoliposomes open so that they show single-layered membrane patches having a height of 5.8 ± 0.7 nm (average ± S.D.; *n* = 28). Scale bar corresponds to 100 nm. Histogram of diameter (**b**) and height (**c**) of PAR1 particles imaged in (**a**). (**b**) The diameter distribution showed two peaks centered at 8.1 ± 1.7 nm (average ± S.D., *n* = 84) and 14.2 ± 4.2 nm. The diameter distribution was fitted with a Gaussian mixture model (red curve) to reveal the average values and S.D. of the peaks. Diameters were measured at full-width half maximum of particle heights. (**c**) The height distribution showed two peaks centered at 1.2 ± 0.4 nm (average ± S.D., *n* = 84) and 2.6 ± 0.8 nm. The height distribution was fitted with a Gaussian mixture model (red curve) to reveal the average values and S.D. of the peaks. (**d**) Single PAR1 embedded into the atomistic model of a DOPC membrane. The model shows the PAR1 (PDB ID 3VW7)[1] protruding ≈1.4 nm from the extracellular lipid membrane surface and protruding ≈1.2 nm from the intracellular lipid surface. Because the atomistic model (PDB ID 3VW7) lacks the 90 amino acid (aa) long N-terminus (which was not solved in the crystal structure), the ≈1.4 nm rather reflects a minimum height of the extracellular PAR1 surface. The FD-based AFM topograph was recorded in buffer solution (300 mM NaCl, 20 mM HEPES, 25 mM $MgCl_2$, pH 7.0) at room temperature. *n* gives the number of PAR1 particles analyzed.

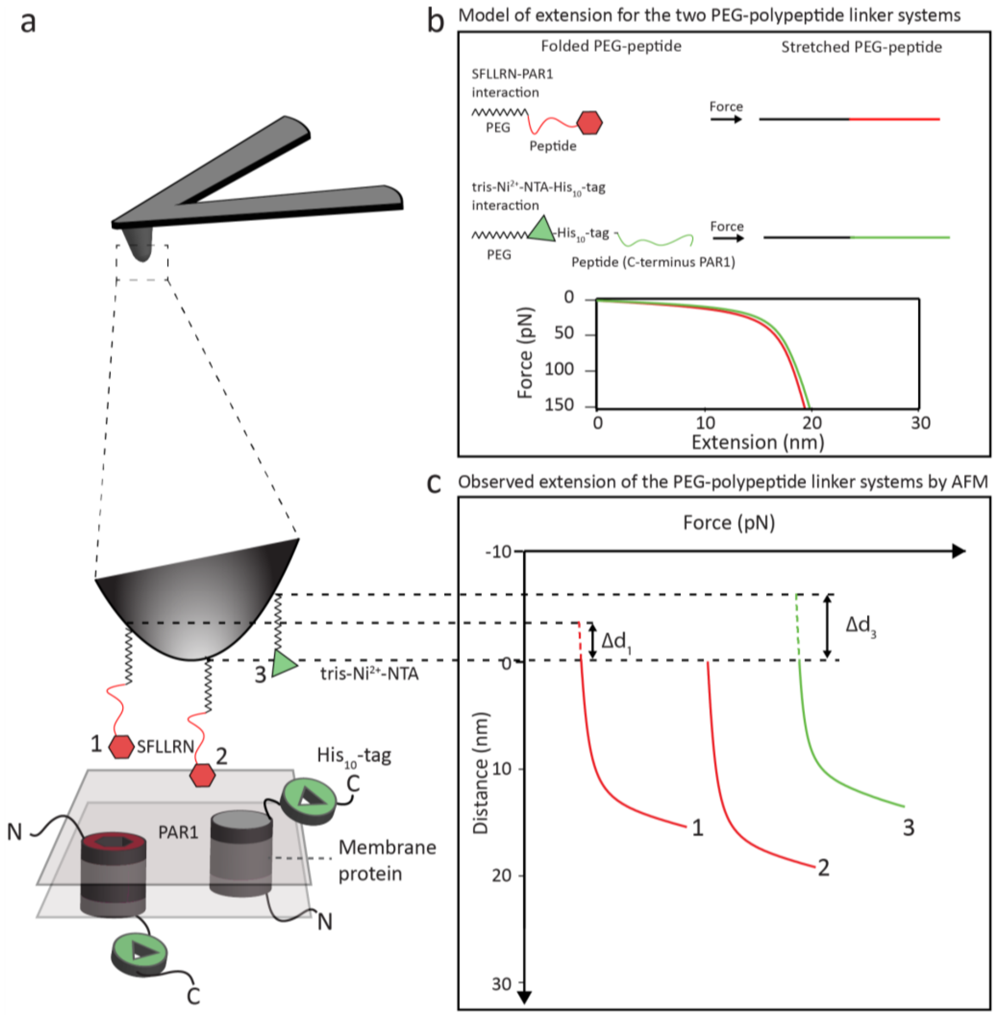

Supplementary Figure 3 | The position of the PEG-polypeptide linker system bound to the AFM tip affects the distance of the force peak stretching the ligand-receptor bond. (**a**) Schematic explaining the different attachment positions of the two linker systems to the AFM tip. PEG-polypeptide linkers 1 and 2 describe the SFLLRN-PAR1 binding. PEG-polypeptide linker 3 describes the binding of the tris-Ni^{2+}-NTA group to the C-terminal His_{10}-tag of PAR1. The stretching of the PEG-polypeptide linker system under force is described combining the model describing the PEG elasticity[2] and the worm-like-chain (WLC) model describing the stretching of the polypeptide[3]. This stretching of the linker system describes the mechanical stressing of the bond formed between the SFLLRN ligand (red extension profile) or the tris-Ni^{2+}-NTA ligand (green extension profile) and PAR1 (**b**). The linker system used to probe the SFLLRN-PAR bond is composed of a 27-unit long PEG (PEG_{27}) spacer and the native N-terminal polypeptide of PAR1 followed by the SFLLRN peptide ligand (sequence: SFLLRN-PNDKYEPFWEDEEKNES-GLTEYRGGGGC). The linker system used to probe the tris-Ni^{2+}-NTA-His_{10}-tag bond is composed of a PEG_{27} spacer, the tris-Ni^{2+}-NTA group attached to the end of the spacer, and the His_{10}-tag attached to the end of the 30 aa long PAR1 C-terminus (SSECQ-RYVYSIL-CCKESS-DPSSYE-NLYFQG-HHHHHHHHHH). Independent of the position at which the PEG-polypeptide linker is chemically anchored to the AFM tip the force-distance characteristics describing the stretching of the different linker systems is similar (Supplementary Note 1). (**c**) Due to the vertical position at which the a linker anchors to the tip, the first part of the force-distance characteristics describing the stretching of the PEG-polypeptide linker system can be hidden by relative distance "Δ*d*" value. Here, we compare three different attachment positions (1, 2, and 3 in (**c**)) of the linker system to the AFM tip.

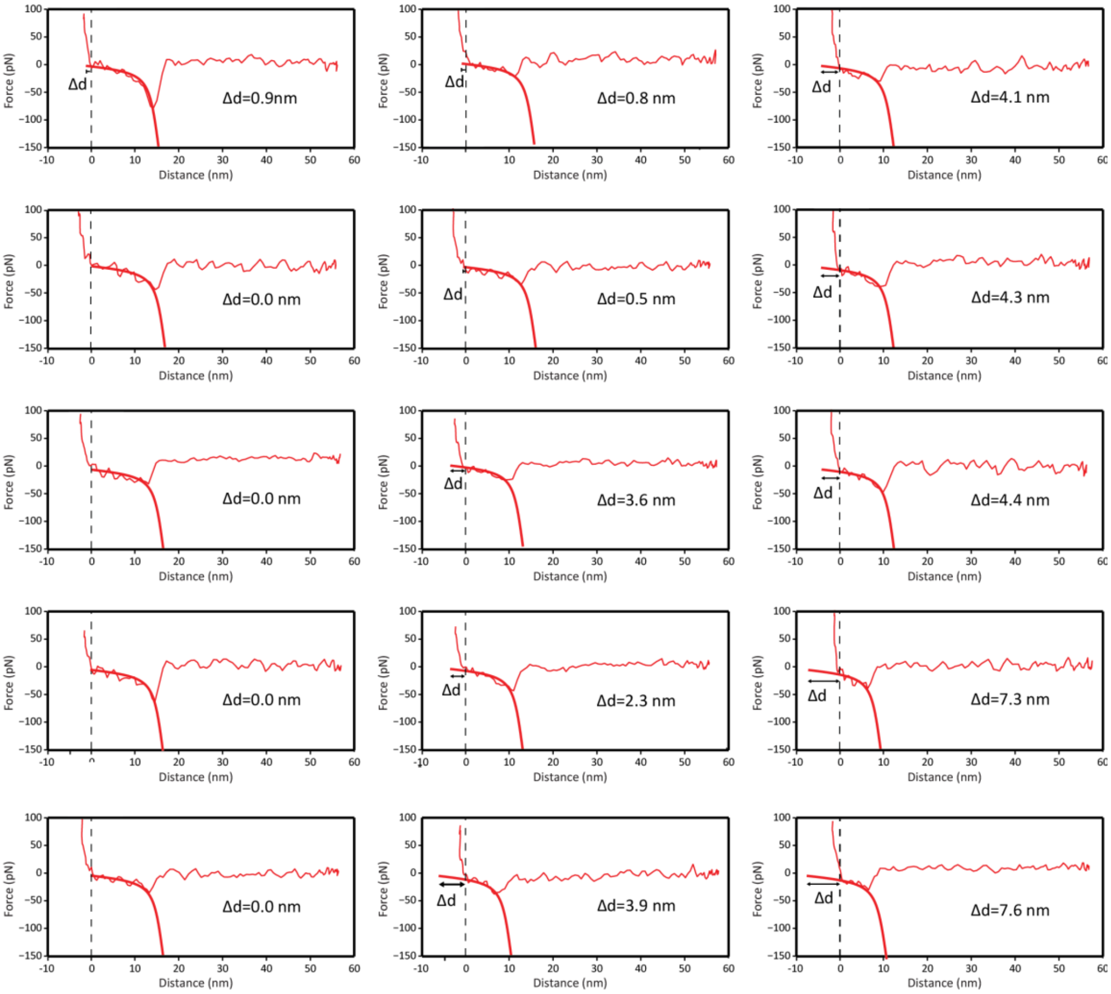

Supplementary Figure 4 | Validating the extension profile of the PEG-polypeptide linker system attaching the SFLLRN ligand to the AFM tip. Shown are individual FD curves, each one detecting the rupture of a specific SFLLRN-PAR1 bond. Each FD curve was fitted using the calculated extension profile of the cantilever-PEG$_{27}$-polypeptide (28 aa) linker system (Supplementary Note 1). The individual fits were shifted along the distance axis to minimize the sum of squared residuals between the fit and the rupture force peak. The Δ*d* values given correspond to the shifted distance. FD curves were recorded as described (Fig. 2) using a SFLLRN functionalized AFM cantilever and imaging a PAR1 proteoliposome by FD-based AFM. The representative force-distance curves were extracted from more than three FD-based AFM maps each one recorded from a new sample preparation and functionalized AFM tip.

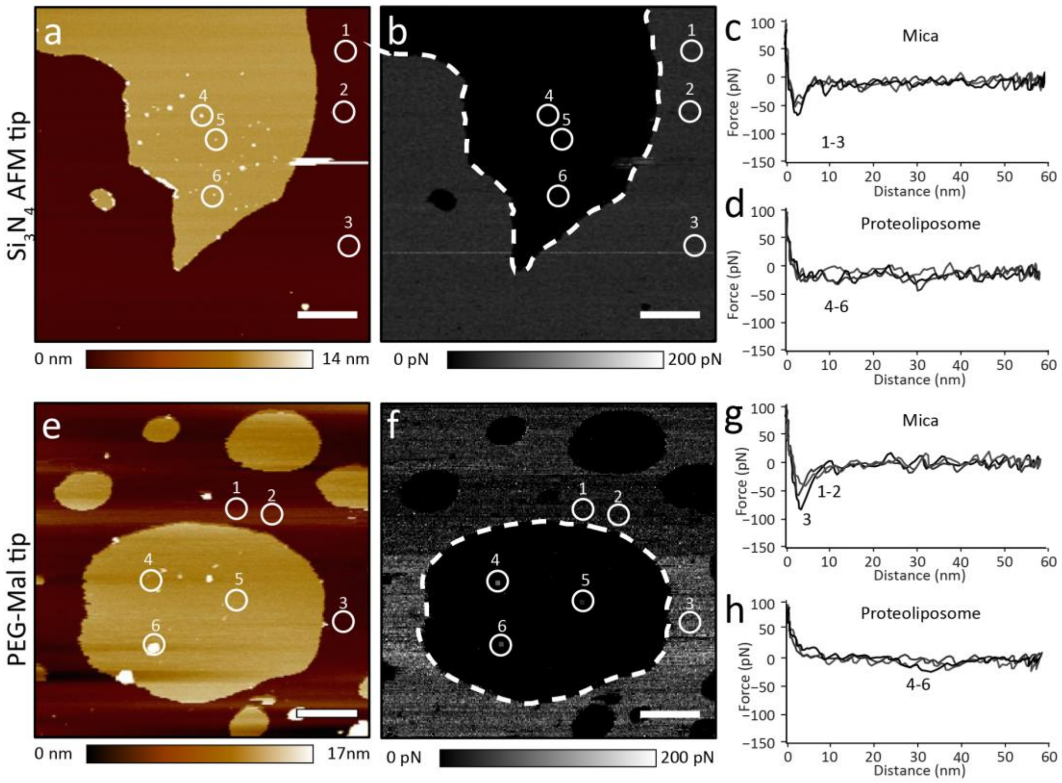

Supplementary Figure 5 | FD-based AFM imaging of PAR1 proteoliposomes using non-functionalized and PEG$_{27}$-maleimide functionalized AFM tips. (**a**) AFM topograph of a PAR1 proteoliposome recorded using a non-functionalized AFM tip. (**b**) Adhesion map simultaneously recorded with the topograph (**a**) showing low unspecific adhesive forces (≈20–60 pN) between the AFM tip and the mica and even lower unspecific adhesive forces (< 30 pN) between the AFM tip and the proteoliposome. (**c**) FD curves showing unspecific adhesion events on mica (FD curves 1–3 were taken at positions 1–3 marked in (**a**) and (**b**)). Characteristically, such unspecific interaction events showed adhesive forces at small tip-sample distances (< 10 nm). (**d**) FD curves showing no adhesion events on the proteoliposome (FD curves 4–6 were taken at positions 4–6 marked in (**a**) and (**b**)). (**e**) AFM topograph of a PAR1 proteoliposome recorded using a PEG$_{27}$-maleimide functionalized tip. (**f**) The adhesion map shows adhesive forces between the functionalized AFM tip and mica (30–180 pN) and no adhesive forces on the proteoliposome. Thus, the PEG$_{27}$-maleimide functionalized tip shows no adhesive interactions with the PAR1 proteoliposome. (**g**) FD curves recorded on mica (FD curves 1–3 were taken at positions 1–3 marked in (**e**) and (**f**)). (**f**) FD curves recorded on the proteoliposome (FD curves 4–6 were taken at positions 4–6 marked in (**e**) and (**f**)). Each experimental condition was repeated at least three times, each time using a new sample preparation and AFM tip. Scale bars correspond to 500 nm.

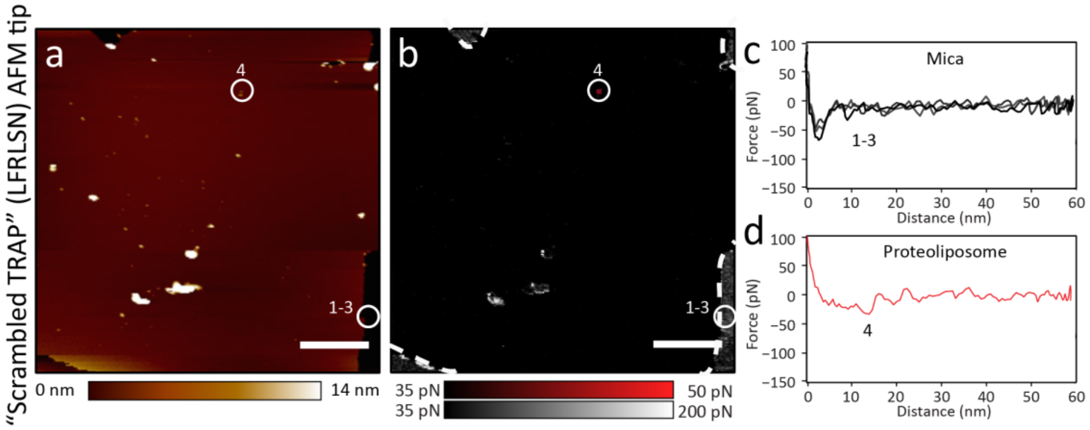

Supplementary Figure 6 | FD-based AFM imaging of PAR1 proteoliposomes using AFM tips functionalized with the scrambled SFLLRN ligand LFRLSN. (**a**) Topography and corresponding (**b**) adhesion map recorded with an AFM tip that has been functionalized with the N-terminal PAR1 sequence ending with a scrambled LFRLSN sequence. For better visibility the pixel of the specific interaction has been enlarged. (**c**) FD curves showing non-specific interactions on mica. (**d**) After repeatedly recording the same proteoliposome four times only one FD curve could be recorded that showed a very weak in the specific region (distance > 7 nm)**.** This control experiment was repeated three times using several functionalized AFM tips with each repetition providing the same result. The FD-based AFM data was recorded as described (Online Methods). Scale bars correspond to 500 nm.

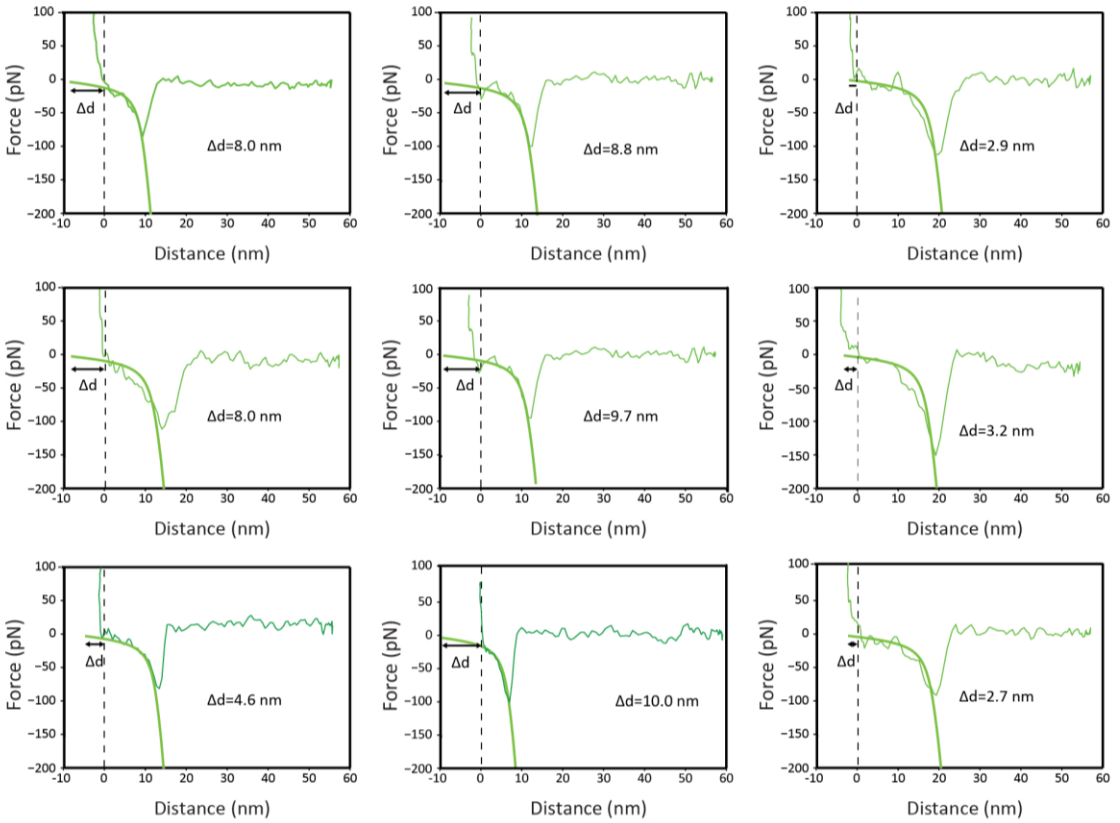

Supplementary Figure 7 | Validating the extension profile of the PEG-polypeptide linker attaching the tris-**Ni^{2+}-NTA ligand to the AFM tip.** Shown are individual FD curves, each one detecting the rupture of a specific SFLLRN-PAR1 bond. Each FD curve was fitted using the calculated extension profile of the cantilever-PEG-(30 aa) polypeptide linker system (Supplementary Note 1 and Supplementary Fig. 3). The individual fits were shifted along the distance axis to minimize the sum of squared residuals between the fit and the adhesion force peak. The Δ*d* values given correspond to the shifted distance. FD curves were recorded as described (Fig. 2) using a tris-Ni^{2+}-NTA functionalized AFM cantilever and imaging a PAR1 proteoliposome by FD-based AFM. The representative FD curves were extracted from more than three FD-based AFM maps each one recorded using a new sample preparation and functionalized AFM tip.

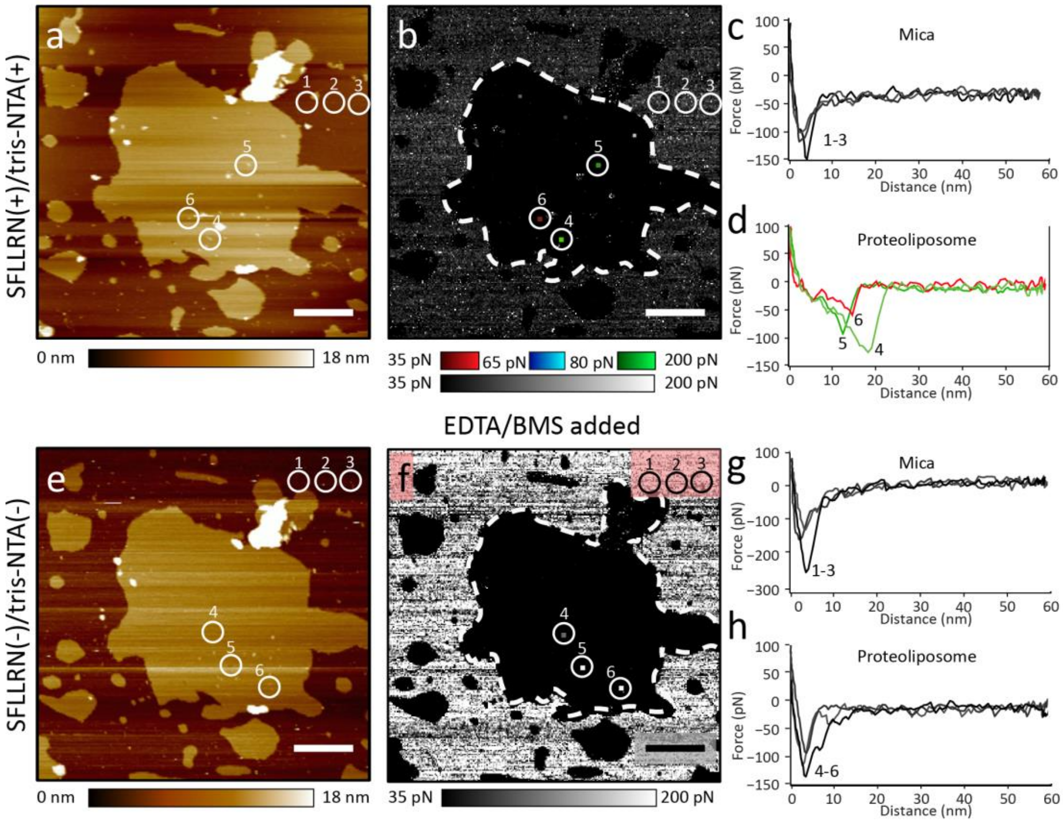

Supplementary Figure 8 | Blocking the specific ligand binding interactions of the bi-functionalized AFM tip using EDTA and BMS. (**a**) FD-based AFM topograph of PAR1 proteoliposomes recorded with the AFM tip bi-functionalized with active tris-NTA (tris-NTA(+)) and SFLLRN (SFLLRN(+)) ligands. Measurements were performed in the presence of Ni^{2+}-ions to support the formation of the tris-Ni^{2+}-NTA-His_{10}-tag bond. (**b**) Adhesion map generated from FD curves recorded while recording the topograph shown in (**a**). (**c**) Example of FD curves detecting unspecific interactions of the bi-functionalized AFM tip with mica (FD curves 1–3 were taken at positions 1–3 marked in (**a**) and (**b**)). (**d**) Example of an FD curve detecting specific interactions of the TRAP-PAR1 bond (FD curve 6 was taken at position 6 marked in (**a**) and (**b**)) and of the tris-Ni^{2+}-NTA-His_{10}-tag bond (FD curves 4 and 5 were taken at positions 4–5 marked in (**a**) and (**b**)). Each of the positions 4–6 co-localized with PAR1s in proteoliposomes. (**e**) AFM topograph of the proteoliposome imaged in (**a**) recorded when the specific interactions of both ligands functionalizing the AFM tip were blocked. BMS was added to the imaging buffer to suppress specific interactions of the SFLLRN ligand (SFLLRN(-)), and EDTA was added to suppress specific interaction of the tris-NTA ligand ((tris-NTA(-)). (**f**) Adhesion map of the topograph recorded in (**e**). (**g**) Example of FD curves showing unspecific interactions recorded on mica (FD curves 1–3 were taken at positions 1–3 marked in (**e**) and (**f**)). (**h**) Example of FD curves showing unspecific adhesion events recorded on the proteoliposome (FD curves 4–6 were taken at positions 4–6 marked in (**e**) and (**f**)). Images were recorded in imaging buffer (300 mM NaCl, 20 mM HEPES, 25 mM $MgCl_2$, pH 7.2) and as stated in the presence of 5 mM $NiCl_2$, 2 µM BMS and/or 10 mM EDTA. For better visibility pixels detecting adhesion events have been enlarged. Each experiment was repeated at least three times, each time the experiment was recorded from a new sample preparation and using a newly functionalized AFM tip. Scale bars correspond to 500 nm.

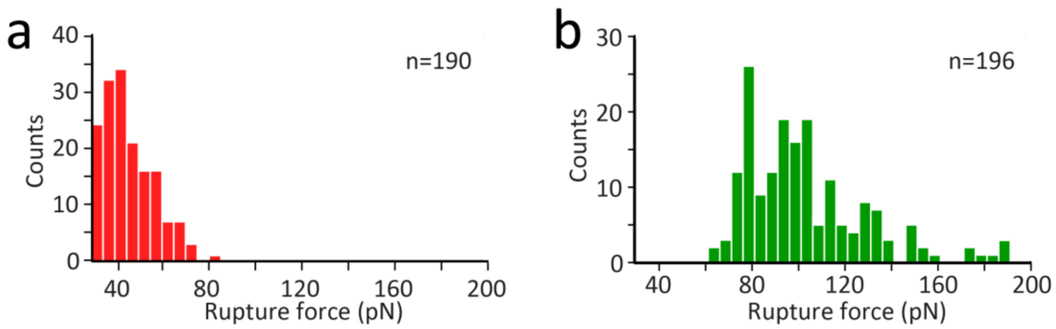

Supplementary Figure 9 | Recording consecutive topographs of the same proteoliposome increase the number of rupture events detected of single SFLLRN-PAR1 and tris**-**Ni^{2+}**-NTA-**His_{10}**-tag bonds.** (**a**) Distribution of forces characterizing only the rupture of single SFLLRN-PAR1 bonds. To prevent formation of the tris-Ni^{2+}-NTA-His_{10}-tag bond, the FD-based AFM data was recorded in the absence of Ni^{2+}. (**b**) Distribution of forces characterizing only the rupture of single tris-Ni^{2+}-NTA-His_{10}-tag bonds. The rupture forces shown were taken from FD-based AFM data recorded using an AFM tip bi-functionalized with tris-NTA and SFLLRN ligands. To support formation of tris-Ni^{2+}-NTA-His_{10}-tag bonds, the FD-based AFM data was recorded in the presence of $NiCl_2$ and to suppress the formation of SFLLRN-PAR1 bonds the antagonist BMS was added. Data was recorded in imaging buffer (300 mM NaCl, 20 mM HEPES, 25 mM $MgCl_2$, pH 7.2) and as stated in the presence of 2 µM BMS and/or 5 mM $NiCl_2$. The rupture forces were collected from eight different topographs and adhesion maps recorded from the same PAR1 proteoliposome.

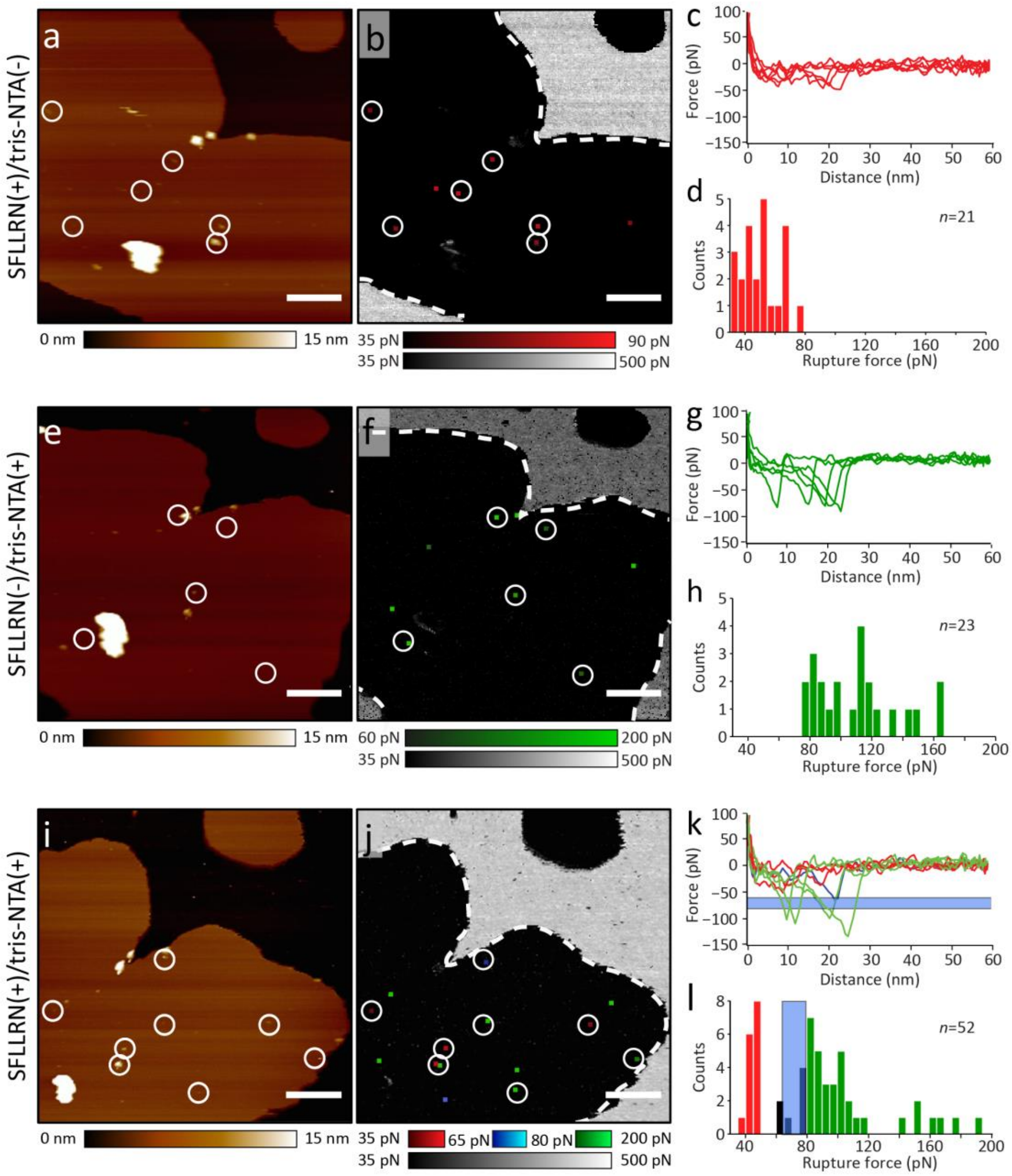

Supplementary Figure 10 | Additional data set: Imaging PAR1 proteoliposomes using bi-functionalized AFM tips and mapping the binding of SFLLRN or tris-NTA ligands. (**a-d**) Imaging the proteoliposome and mapping the binding of the SFLLRN ligand to PAR1. (**a**) Topograph of a PAR1 proteoliposome recorded with inactivated tris-NTA (tris-NTA(-)) and active SFLLRN (SFLLRN(+)) ligand. Experiments were conducted in the absence of Ni^{2+} allowing only the SFLLRN peptide to bind to PAR1. (**b**) Adhesion map showing specific rupture forces (circles) on the proteoliposome (dashed) imaged in (**a**). (**c**) FD curves recording specific interactions in (**b**). (**d**) Rupture forces of single SFLLRN-PAR1 bonds range from 35–80 pN, collected from three repetitive recordings. (**e-h**) Imaging the proteoliposome and mapping the binding of the tris-NTA ligand to the intracellular His_{10}-tag of PAR1. (**e**) Topograph recorded with activated tris-NTA (tris-NTA(+)) and blocked SFLLRN (SFLLRN(-)) ligand in the presence of Ni^{2+} and the antagonist BMS. (**f**) Adhesion map showing specific rupture forces (circles) on the proteoliposome (dashed) imaged in (**e**). (**g**) FD curves recording specific interactions in (**f**). (**h**) Rupture forces of single tris-NTA-His_{10}-tag bonds range from 65–140 pN, collected from three repetitive recordings. (**i-l**) Imaging the proteoliposome and mapping the interaction of SFLLRN-PAR1 and tris-NTA His_{10}-tag bonds. (**i**) Topograph recorded with active tris-NTA (tris-NTA(+)) and active SFLLRN (SFLLRN(+))

ligands. Measurements were performed in the presence of Ni^{2+} to allow both tip-tethered ligands to interact with PAR1. (**j**) Adhesion map showing specific rupture forces (circles) on the proteoliposome (dashed) imaged in (**i**). (**k**) FD curves showing specific adhesion forces recorded in (**j**). (**l**) Rupture forces of SFLLRN-PAR1 (red) and of tris-NTA-His_{10}-tag (green) bonds recorded in three consecutive recordings. A force filter from 65–80 pN (blue) discriminates between both types of specific interactions (red *vs.* green). (**d,h,l**) Rupture force distributions. Experiments were recorded in buffer solution (300 mM NaCl, 20 mM HEPES, 25 mM $MgCl_2$, pH 7.2) and if stated 2 µM BMS or/and 5 mM $NiCl_2$ were added. Pixels detecting adhesion events were enlarged. Each experiment was repeated at least three times preparing a new sample and using a newly functionalized AFM tip. Scale bars, 500 nm.

Supplementary Note 1

The extension of the 'PEG-polypeptide linker system' can be calculated by the combination of a worm-like-chain (WLC) model[3,4] describing the peptide extension using a persistence length of 0.4 nm per amino acid and a the model for PEG elasticity[2] in water to describe the 27 (n_m) PEG spacer (PEG_{27}) extension. To calculate the PEG_{27} spacer extension we have used a Kuhn length of 0.7 nm (L_k), a segment elasticity (K_s) of 150 nN nm^{-1}, a monomer length in planar configuration of 0.358 nm (L_{planar}), a monomer length in helical configuration of 0.28 nm ($L_{helical}$) and a free energy difference between planar and helical configuration of $\Delta G = 3\ k_b T$.

Worm-like-chain model[3,4]:

$$F = \frac{k_b T}{l_P}\left(\frac{1}{4\left(1 - \frac{x}{L_c}\right)^2} + \frac{x}{L_c} - 0.25 \right)$$

Model for PEG elasticity[2]:

$$L(F) = L_C(F) \times \left(\coth\left(\frac{F l_k}{k_b T}\right) - \frac{k_b T}{F l_k} \right) + \frac{n_m F}{K_s}$$

$$L_C(F) = n_m \times \left(\frac{L_{planar}}{e^{\frac{\Delta G(F)}{k_b T}} + 1} + \frac{L_{helical}}{e^{\frac{-\Delta G(F)}{k_b T}} + 1} \right)$$

$$\Delta G(F) = \Delta G - F\left(L_{planar} - L_{helical}\right)$$

Combining both models describing the polypeptide and PEG stretching, we obtained the force-extension relationship of the PEG-polypeptide linker system for either measuring the SFLLRN-PAR1 bond or the tris-Ni^{2+}-NTA-His_{10}-tag bond (Supplementary Fig. 3). When analyzing all recorded interaction force curves we could correlate the force curves detecting the rupture of the SFLLRN-PAR1 bond or the tris-Ni^{2+}-NTA-His_{10}-tag bond with their respective extension profile (Supplementary Figs. 4 and 7). A few (≤ 5%) of the force peaks detecting the rupture of the SFLLRN-PAR1 bonds occurred at a longer distance than expected (>>30 nm). This shift towards longer distances can be explained by lifting the soft lipid bilayer from the supporting mica[5]. On the contrary, when analyzing the rupture force peaks of tris-Ni^{2+}-NTA-His_{10}-tag interactions recorded from the intracellular PAR1 surface we observed comparatively more (≈15%) extension profiles that were slightly larger than the stretched PEG_{27}-30 aa linker system. Due to the higher interaction forces needed to rupture the tris-Ni^{2+}-NTA-His_{10}-tag bond it might be that the soft lipid bilayer lifted more frequently from the supporting mica. It may be also speculated that upon direct exposure to the rupture force the C-terminal alpha-helix of PAR1 unfolded partly. It has been frequently reported that transmembrane helices of GPCRs can unfold at forces of < 100 pN[6-8]. This unfolding of a transmembrane helix extends the length of the linker system by the portion of the unfolded polypeptide (up to ≈25 aa)[9,10]. However, in the few cases in which rupture force peaks were detected at too long distances (>>30 nm, see Results), which could not be explained by the length of the linker system, they were discarded from further analysis.

Supplementary References

1. Zhang, C. et al. High-resolution crystal structure of human protease-activated receptor 1. *Nature* **492**, 387-92 (2012).
2. Oesterhelt, F., Rief, M. & Gaub, H.E. Single molecule force spectroscopy by AFM indicates helical structure of poly(ethylene-glycol) in water. *New J Phys* **1**, 6 (1999).
3. Bustamante, C., Marko, J.F., Siggia, E.D. & Smith, S. Entropic elasticity of lambda-phage DNA. *Science* **265**, 1599-600 (1994).
4. Rief, M., Gautel, M., Oesterhelt, F., Fernandez, J.M. & Gaub, H.E. Reversible unfolding of individual titin immunoglobulin domains by AFM. *Science* **276**, 1109-1112 (1997).
5. Kocun, M. & Janshoff, A. Pulling tethers from pore-spanning bilayers: towards simultaneous determination of local bending modulus and lateral tension of membranes. *Small* **8**, 847-51 (2012).
6. Park, P.S. et al. Stabilizing effect of Zn2+ in native bovine rhodopsin. *J Biol Chem* **282**, 11377-85 (2007).
7. Kawamura, S. et al. Kinetic, energetic, and mechanical differences between dark-state rhodopsin and opsin. *Structure* **21**, 426-37 (2013).
8. Zocher, M., Zhang, C., Rasmussen, S.G., Kobilka, B.K. & Muller, D.J. Cholesterol increases kinetic, energetic, and mechanical stability of the human beta2-adrenergic receptor. *Proc Natl Acad Sci U S A* **109**, E3463-72 (2012).
9. Oesterhelt, F. et al. Unfolding pathways of individual bacteriorhodopsins. *Science* **288**, 143-6 (2000).
10. Engel, A. & Gaub, H.E. Structure and mechanics of membrane proteins. *Annu Rev Biochem* **77**, 127-48 (2008).
